# Memory Profiles after Unilateral Paramedian Thalamic Stroke Infarction: A Comparative Study

**DOI:** 10.1155/2015/430869

**Published:** 2015-10-26

**Authors:** Antonio Carota, Herbert Neufeld, Pasquale Calabrese

**Affiliations:** ^1^Division of Molecular and Cognitive Neuroscience, Neuropsychology and Behavioural Neurology Unit, Faculty of Psychology and Interdisciplinary Platform Psychiatry and Psychology, University of Basel, 4055 Basel, Switzerland; ^2^GSMN Neurocenter, Genolier Clinic, Genolier Swiss Medical Network, 1272 Genolier, Switzerland

## Abstract

We performed extensive neuropsychological assessment of two male patients (matched for age and educational level) with similar (localization and size) unilateral paramedian ischemic thalamic lesions (AB on the left and SD on the right). Both patients showed severe memory impairments as well as other cognitive deficits. In comparison to SD, AB showed severe impairment of executive functions and a more severe deficit of episodic/anterograde memory, especially in the verbal modality. The findings of this single case study suggest the possibility that the profile and severity of the executive dysfunction are determinant for the memory deficits and depend on from the side of the lesion. In addition to a material-side-specific (verbal versus visual) deficit hypothesis, the differential diencephalo-prefrontal contributions in mnestic-processing, in case of paramedian thalamic stroke, might also be explained in terms of their stage-specificity (encoding versus retrieval).

## 1. Introduction

Despite several well-documented case reports and several group studies on the cognitive deficits due to thalamic damage [1, 2], there are still some questions regarding the role of the anterior and paramedian thalamic nuclei and related bundles in memory processing. A persistent amnesic syndrome is generally the consequence of bilateral lesions of these nuclei; however, a severe memory disturbance might result from unilateral thalamic damage [3, 4]. Whether only material-specific memory deficits may occur as the result of unilateral lesions is debated. If the pervasive amnesic syndrome is also found in the case of unilateral lesions, different factors may be responsible [5] such as a more or less relevant role of frontal-thalamic functional systems.

In the last decades, other memory classifications regarding content-related components, like episodic and semantic memory, were established, but these classifications did not take into consideration different neural processing between verbal and visual aspects of the episodic anterograde memory [6]. While limbic structures, including thalamic nuclei, are postulated to be more involved in encoding and storage, prefrontal regions are supposed to be more critical for retrieval [6]. Executive dysfunction can be particularly prominent after ischemic lesions of the dorsomedial nucleus (paramedian thalamic stroke) [7, 8]. The dysexecutive syndrome, occurring in these cases, includes loss of mental flexibility and reasoning, confabulations, and frontal-type memory impairments, such as a defective retrieval strategy [9, 10]. Van Der Werf and coworkers [4] described amnestic deficits in both the visual and verbal modalities in a patient with only right thalamic damage. This patient also showed dysexecutive deficits and the authors hypothesized that the cognitive profile of deficits was completely the result of the dysexecutive syndrome due to the interruption of thalamocortical connections.

Other authors discussed cognitive profiles based on exact anatomical lesion analysis depending on the damage of distinct substructures within the thalamus [7, 11, 12]. Thus, more extensive neuropsychological assessments and comparative analyses of the cognitive profiles of different thalamic lesions would be helpful in understanding the nature of memory deficits with respect to the influence of the disruption of functional neural systems connected to specific thalamic nuclei.

We had the opportunity to study two patients with similar unilateral paramedian thalamic stroke. One of them showed a lesion limited to the left paramedian thalamic region while for the other patient the contralateral homologous region of the thalamus was damaged with extension to the mesencephalon. The lesions were comparable (even if not completely) for anatomical localization and size. The aim of our study was to study the cognitive profiles of the two patients, who differentiated only for the side of the lesions, in order to delineate specific factors influencing memory performances.

## 2. Case Reports

### 2.1. Case  1

**Figure 1:**
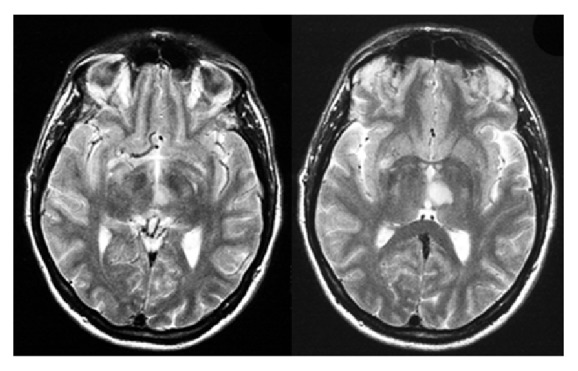
Transversal brain MRI (FLAIR) images of the patient AB showing the acute ischemic lesion in the medial dorsal nucleus of the left thalamus.

A 45-year-old right-handed male insurance maker (AB) with 12 years of education and unremarkable medical history presented with somnolence and disorientation with regard to time and location, without lateralizing signs at the neurological examination. Two weeks after admittance he improved in orientation. MRI was performed one day after admission and revealed an infarction in the left paramedian thalamus (dorsomedial nucleus) (Figure 1). Detailed neuropsychological examination was performed 3 weeks after admission.

### 2.2. Case  2

**Figure 2:**
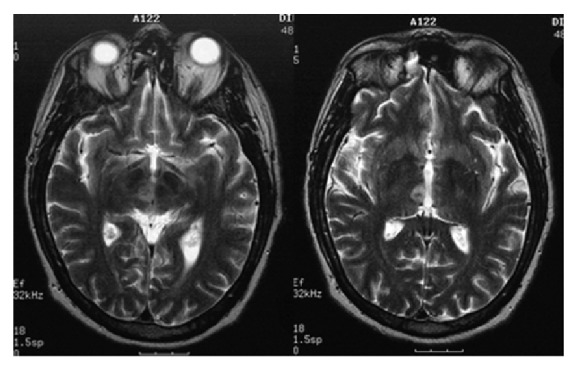
Brain MRI (T2 weighted) of the patient SD showing the acute ischemic lesion confined to the right paramedian thalamus extending to the mesencephalic tegmentum.

A 49-year-old right-handed male real estate manager (SD) with 12 years of education and a medical history of arterial fibrillation and hypertension was admitted to the hospital after sudden onset of diplopia, dysarthria, and temporal and topographical disorientation. Neurological examination showed vertical left internuclear ophthalmoplegia without lateralizing signs. During the next five days, speech and orientation improved. A brain MRI was effectuated one week after stroke and showed an ischemic lesion confined to the right paramedian thalamus extending to the mesencephalic tegmentum (Figure 2). Detailed neuropsychological examination was performed 3 weeks after admission.

Both subjects had comparable sociocultural backgrounds.

## 3. Neuropsychological Examination

We tested AB and SD with a number of neuropsychological measures in order to evaluate the patients' level of intelligence, attention and concentration, memory performance, and executive functions. The results are summarised in Table 1.

### 3.1. Intelligence

Intellectual abilities were examined with the shortened German version of the Wechsler Adult Intelligence Test including the following subtests: general information, similarities, picture completion, and block design. Both patients showed a comparable level of average intellectual performance, displaying an IQ slightly above 100, a score that was concordant with their educational level.

### 3.2. Attention and Concentration

Attention was evaluated with a computerised attention test battery (TAP) [13]. Alertness was assessed through reaction times of responses to a cross in the centre of a screen (tonic alertness, condition A). Half of the trials (condition B) were preceded by a tone serving as a warning cue. All the trials (80 altogether) were arranged in an ABBA-design, allowing calculation of an alerting factor by subtracting the mean reaction times of trials A from trials B. Divided attention was evaluated through parallel presentation of visual and acoustic stimuli during which the patient had to react to certain stimuli configurations. AB performed below average performance while the results of SD regarding tonic and phasic alertness were within normal limits. SD's divided attention performance was impaired.

### 3.3. Executive Functions

The Wisconsin Card Sorting Test (checking concept formation and tendency to perseveration) revealed that AB showed a significant number of perseverative as well as nonperseverative errors and poor performance in concept formation. His performance in the Tower of Hanoi Test revealed considerable deficits in planning and problem solving. SD showed normal performance in both tests.

### 3.4. Anterograde Memory

By means of the revised Wechsler Memory Scale, verbal and visual anterograde memory was examined. This large memory test battery includes different kinds of tasks, like recognition, free and cued recall, and short-term and working memory tests for verbal as well as visual material. The general memory index was below average in both patients. AB and SD also scored below the average range for the verbal memory index. The visual memory index was normal for AB, but not for SD. In the delayed recall condition, AB performed far below average, while SD's delayed recall index was within the lower average range. Both patients showed nearly normal short-term memory performance; however working memory was impaired for both of them. Visual and verbal paired-associate learning was significantly poor for both patients. In the logical memory subtest (story recall) poor performance in immediate condition was observed in both patients, whereas in the delayed condition only SD showed a normal performance. In the immediate reproduction of simple drawings AB performed in the normal range but his results were below average in the delayed condition, whereas SD showed an opposite pattern. The Rey Auditory-Verbal Learning Test was performed to measure abilities of verbal list-learning and delayed recall. AB showed poor results in verbal learning and was not able to remember any word at all in the delayed recall condition. SD on the other hand showed an average verbal learning ability and normal results in delayed recall for words. Visual delayed reproduction for complex information was examined also with the Rey-Osterrieth Figure. AB scored below average in this condition, while SD showed normal delayed visual memory ability.

### 3.5. Visual-Spatial Performance

Visual-spatial performance was examined with the copy of the Rey-Osterrieth Figure and block tapping design. Under the two conditions, AB and SD showed no impairments.

### 3.6. Mood and Affect

In order to measure a possible tendency towards depression, the total score of the Beck Depression Inventory was calculated. Both patients scored below the critical cut-off value, which seems to discard the role of depressive symptoms for the results of memory tests and the whole cognitive evaluation.

## 4. Discussion

Comprehensive neuropsychological assessment of our two patients confirmed the material-side-specific processing hypothesis of the thalamic anterograde memory system. Patient AB, who suffered a left paramedian thalamic stroke, had more pronounced deficits in the verbal modality while the deficit of patient SD with a right thalamic lesion was prominent in the visuospatial domain. However, although AB's performance in the immediate visual recall condition was close to average, his delayed recall was extremely poor in both modalities. On the other hand, SD showed a somewhat better performance in the immediate verbal task, while his delayed recall was within normal limits in both conditions. Thus, our results may be interpreted on the basis of level-specific failure of memory processing. Firstly, both patients showed severe difficulties in learning stimuli, a finding that may be interpreted in the sense of reduced encoding. As suggested by Mayes and Downes [14] encoding can be seen as a process that leads to the formation of representations that may or may not be stored in memory. Secondly, AB's accentuated rate of forgetting points to an additional storage impairment since his poor delayed free as well as cued recall performance in both modalities cannot be attributed only to impaired retrieval. Several authors who have found prefrontal dysfunction in amnesic patients with unilateral thalamic damage imputed additional deficits of the executive functions to be responsible for amnesia [4, 5, 9]. These findings raise the question of the influence of prefrontal disturbances in memory processing. One argument would be that pervasive amnesia can be caused by defective retrieval strategies, which per se depend critically on the functional integrity of prefrontal structures. This assumption is not corroborated by the findings of our study, as we did not find any disproportionate deficit between free recall and cued recall measures. Indeed, we suggest that the integrity of prefrontal circuitries could be crucial at the encoding and storage levels of information processing. Thus, contrary to the retrieval-based explanation, we propose an alternative view, by assuming that AB's substantial encoding and storage deficits would be the consequences of a more extended left thalamic lesion, which includes the internal medullary lamina and the mammillothalamic tract. As suggested by von Cramon and coworkers [11], the combined damage of the intralaminar region and mammillothalamic tract might be the critical dysfunction for memory consolidation processes. Such a combined lesion could involve bottleneck-structures of the medial and basolateral limbic loop. Furthermore, the larger paramedian thalamic stroke in AB probably interrupted the chain of information flow between diencephalon and prefrontal cortex, leading to a disconnection-syndrome characterised both by executive dysfunction and amnesia.

Additional support for an encoding and storage based deficit on the basis of a combined diencephalo-prefrontal damage stems from several neuroimaging studies, which demonstrated an asymmetrical involvement of the prefrontal region in different stages of memory processing [15, 16]. These studies showed that while the left prefrontal cortex is more engaged in the encoding process of memory traces, the right prefrontal cortex would be more activated during the retrieval process. In most reported cases on prefrontal disturbances after unilateral thalamic stroke, dysfunction was found on the ipsilateral side of the frontal lobe [17, 18]. Thus, in the case of the left sided thalamic damage in AB, it can be assumed that his executive impairment is more likely to stem from the dysfunction of the ipsilateral left prefrontal region. According to the aforementioned hypothesis regarding asymmetrical involvement of the prefrontal region in encoding and retrieval processes, more difficulties in early stages of processing would be expected in patients with left sided lesions while more retrieval difficulties would arise after right sided lesions. Again, this is in agreement with the findings of the patient AB. Van Der Werf and coworkers [4] reported a patient with a right sided thalamic lesion, who showed decreased perfusion of the right prefrontal region. Neuropsychological investigation revealed visual and verbal memory impairments as well as executive disturbances. Based on the patient's cognitive profile the authors suggested that the memory deficits could not be attributed to problems in early stages of information processing and are hence regarded as resulting from a failure of retrieval rather than encoding or storage. This finding is also in agreement with the prefrontal asymmetry hypothesis.

In an elegant study, Mennemeier and colleagues [19] presented the cognitive profile of a patient with a left intralaminar thalamic stroke. The authors could demonstrate that when the patient was able to use semantic encoding strategies, his memory performance was nearly normal. Such strategies are well known to depend on the left prefrontal region, which was spared in the case of that patient. If usage of these strategies was impossible in the verbal tasks, the patient' s performance was worse than in control subjects and thus showed clearly susceptibility to the effects of an interfering task on the Peterson-Peterson paradigm. The analysis of our patient SD's performances in memory tasks provides a similar interpretation. Although showing a generally reduced encoding capacity he performed nearly normal in all delayed recall tasks except in the semantically unrelated word list of the auditory-verbal learning task. In this task, which is not suited for semantic clustering, SD's retroactive interference level was significantly increased and his delayed recall was poor. In other tasks, his delayed performance in free recall as well as cued recall condition was within lower normal limits. On the contrary, AB's insufficient performance in delayed memory tasks might result from his inability to use semantic encoding strategies due to the left prefrontal dysfunction. Semantic failure for object-recall could be prominent with left thalamic stroke [20].

In conclusion, our findings seem to indicate that a diencephalo-prefrontal disconnection probably causes the prefrontal disturbances occurring in patients with unilateral thalamic damage. However, this disconnection could differentially contribute to the profile of memory deficits depending on the size and the site of lesion. Finally, we propose that, in addition to a modality (verbal versus visual) deficit hypothesis (dependent on the lesion-side), the differential diencephalo-prefrontal contributions in mnestic-processing in case of paramedian thalamic stroke should also be explained in terms of their stage-specificity (encoding versus retrieval).

## Figures and Tables

**Table 1:** Comparison of AB and SD performances for neuropsychological tests. Normative data are indicated as mean and standard deviation, raw scores, or percentiles. Insufficient values are indicated in bold characters.

Test/score	AB	SD	Norms
*Intelligence*			
Reduced Wechsler Intelligence Test			
IQ	104	109	100 ± 15
*Attention/concentration*			
Wechsler Memory Scale-revised (WMS-R)			
Attention/concentration index	76	74	100 ± 15
Computerised attention test battery			
Tonic alertness without warning tone	**5%le**	46%le	>15%le
Tonic alertness with warning tone	**4%le**	69%le	>15%le
Phasic alertness	21%le	82%le	>15%le
Divided attention	**1%le**	**7%le**	>15%le
*Executive function*			
Wisconsin Card Sorting Test (Nelson form)			
Nonperseverative errors	**12**	7	7 ± 5
Perseverative errors	**14**	1	7 ± 5
Categories	**3**	6	5.6 ± 1.1
Tower of Hanoi (3-disc version)			
Number of moves	24	7	7
*Mood and affect*			
Beck Depression Inventory			
Sum score	10	7	<17
*Anterograde memory*			
Wechsler Memory Scale-revised (WMS-R)			
WMS-R indexes			
General memory	**68**	**75**	100 ± 15
Verbal memory	**60**	**76**	100 ± 15
Visual memory	89	**81**	100 ± 15
Delayed recall	**56**	86	100 ± 15
WMS-R subtests			
Digit span, forwards	69%le	34%le	>15%le
Digit span, backwards	**2%le**	**2%le**	>15%le
Block tapping, forwards	**5%le**	50%le	>15%le
Block tapping, backwards	**11%le**	**2%le**	>15%le
Logical memory, immediate	**2%le**	16%le	>15%le
Logical memory, delayed	**3%le**	21%le	>15%le
Visual reproduction, immediate	42%le	19%le	>15%le
Visual reproduction, delayed	**10%le**	27%le	>15%le
Verbal paired associate learning			
Immediate (number of pairs/trials)	**4/6**	**8/6**	8/3
Delayed (number of pairs)	**1**	7	7
Visual paired associate learning			
Immediate (number of pairs/trials)	**6/5**	**4/6**	6/3
Delayed (number of pairs)	**2**	**4**	5
Rey Auditory-Verbal Learning Test			
A5 (fifth)	**6**	11	11
Delayed recall	**0**	**5**	10
Rey-Osterrieth Figure			
Copy	34	36	35
Delayed reproduction	13	22	10
